# Efficacy of Pyrotinib combined with Trastuzumab in the treatment of patients with human epidermal growth factor receptor-2 positive breast cancer

**DOI:** 10.12669/pjms.41.6.11682

**Published:** 2025-06

**Authors:** Bin Tang, Xiuwen Yi, Qinghua Mo, Yulan Tang, Lingling Zhang, Liming Xie

**Affiliations:** 1 Bin Tang Department of Neurology, The First People’s Hospital of Hengyang, Hengyang, Hunan Province 421000, P.R. China; 2 Xiuwen Yi Department of Medical Oncology, Hengyang Medical School, University of South China, The First Affiliated Hospital, Hengyang Medical School, University of South China, Hengyang, Hunan Province 421000, P.R. China; 3 Qinghua Mo Department of Medical Oncology, The First People’s Hospital of Hengyang, Hengyang, Hunan Province 421000, P.R. China; 4 Yulan Tang Department of Medical Oncology, The First People’s Hospital of Hengyang, Hengyang, Hunan Province 421000, P.R. China; 5 Lingling Zhang School of Medical Health Management, Hunan Vocational College of Foreign Languages, Changsha, Hunan Province, P.R. China; 6 Liming Xie Department of Medical Oncology, Hengyang Medical School, University of South China, The First Affiliated Hospital, Hengyang Medical School, University of South China, Hengyang, Hunan Province 421000, P.R. China

**Keywords:** Breast cancer, Human epidermal growth factor receptor-2, Pyrotinib, Positive, Trastuzumab

## Abstract

**Objective:**

To analyze the efficacy of Pyrotinib combined with Trastuzumab in the treatment of human epidermal growth factor-2 (HER-2) positive breast cancer (BC).

**Methods:**

This was a retrospective study that enrolled 150 patients with HER-2 positive BC admitted to Hengyang Medical School, University of South China from January 2021 to October 2023. Among them, 73 patients were treated with Trastuzumab alone (Trastuzumab group), and 77 cases were treated with combined Pyrotinib and Trastuzumab (combined group). Tumor response, tumor marker levels, immune function, and incidence of toxic side effects were compared between the two groups.

**Results:**

Tumor response of the combined group (94.81%) was significantly higher than that of the Trastuzumab group (84.93%) (*P*<0.05). After treatment, the levels of carcinoembryonic antigen (CEA), carbohydrate antigen 153 (CA153), carbohydrate antigen 125 (CA125), carbohydrate Antigen 19-9 (CA19-9), COX-2, DcR3 and Caspase-3 in both groups were significantly reduced compared to pretreatment levels, and were significantly lower in the combined group compared to the Trastuzumab group (all *P*<0.05). After treatment, the levels of CD3^+^, CD4^+^, and CD8^+^ in both groups were significantly reduced compared to before treatment, but the combined group was higher than the Trastuzumab group (all *P*<0.05). There was no significant difference in the incidence of toxic side effects between the groups (*P*>0.05).

**Conclusions:**

Compared with Trastuzumab alone, a combined regimen of Pyrrolitinib and Trastuzumab is equally safe, and improving immune function, reducing the level of tumor markers, and improving tumor response in patients with HER-2 positive BC.

## INTRODUCTION

Breast cancer (BC) accounts for approximately 15% of the total number of female malignant tumors, and is associated with high mortality.^1^ About 15%-20% of all BC cases exhibit overexpression of the human epidermal growth factor-2 (HER-2).^2^,^3^ Studies have shown that HER-2 positivity in BC is associated with worse prognosis and shorter overall survival.^4^,^5^ Therefore, identifying safe and effective treatment for patients with HER-2 positive BC is crucial.^3^–^5^

Targeted drugs, such as Trastuzumab and Pyrotinib, are important therapeutic approaches to treating BC.^2^ Trastuzumab is commonly used and has a good anti-tumor effect. By binding to HER-2, Trastuzumab inhibits tumor metastasis and proliferation, and strengthens immune function.^2^,^6^ Pyrotinib is a novel irreversible tyrosine kinase inhibitor that inhibits HER-1, HER-2, and HER-4, and reduces the formation of HER family homo/heterodimers.^7^–^9^ The aim of this study was to determine the effect of Pyrotinib combined with Trastuzumab in HER-2 positive BC to provide a reference basis for the treatment of HER-2 positive BC.

## METHODS

This was a retrospective study that enrolled 150 patients with HER-2 positive BC admitted to Hengyang Medical School, University of South China from January 2021 to October 2023. Among them, 73 patients were treated with Trastuzumab alone and were assigned to the Trastuzumab group, while 77 patients were treated with Pyrotinib combined with Trastuzumab were assigned to the combined group.

### Ethical Approval:

The ethics committee of our hospital approved this retrospective study with the number: 2024KY151, Dated: November 16^th^, 2024.

### Inclusion criteria:

- Met the diagnostic criteria for BC.^10^
- Positive HER2 defined as immunohistochemistry (IHC) category 3+ or IHC category 2+ with fluorescence in situ hybridization positive.
- The disease stage was from stage IIb to stage IIIb.
- Complete clinical data.
- The Eastern Cooperative Oncology Group (ECOG) score ≤ 2 points.

### Exclusion criteria:

- Patients whose condition worsened during the study period and who needed to adjust the intervention plan.
- Patients with presence of infected individuals in important organs.
- Patients with other malignant tumors.
- Breastfeeding and pregnant women.
- Patients with distant metastasis of the lesion.

### Treatment regimens:

Both groups received chemotherapy with docetaxel (intravenous infusion of 75 mg/m2 docetaxel injection+250 ml physiological saline, completed within 60 minutes, with a cycle of three weeks; dosage reductions were performed in patients who had adverse reactions to the treatment). Patients in the Trastuzumab group received Trastuzumab, with an initial dose of 8 mg/kg + 250 ml physiological saline intravenous infusion, completed within 90 minutes, and maintained at a dose of 6 mg/kg. One course of treatment lasted for three weeks, and four courses of treatment were given in total.

Patients in the Combined group received Pyrotinib maleate orally at a dose of 400 mg/dose, once a day, 30 minutes after meals, in addition to the Trastuzumab group. Each course of treatment lasted for three weeks, and four courses of treatment were given in total.

### Follow up:

Follow-up was conducted by telephone or outpatient review. The follow-up time was 12 months. The follow-up content mainly included the patient’s survival status, verification of the patient’s basic information, treatment effect, and adverse reactions.

### Data collection:

- Baseline data, including age, disease stage, pathological type, ECOG score, family history, and hormone receptor status.
- Treatment response. Treatment response was assessed by Response Evaluation Criteria in Solid Tumors (RECIST).^11^ Complete remission: lesion completely disappears and the remission lasts for ≥4 weeks; Partial remission: lesion shrinks by ≥ 30% and the remission lasts for ≥4 weeks; Stable disease: lesion increases by less than 20% or decreases by less than 30%; Progressive disease: appearance of new lesions or an increase in lesion volume of ≥20%. Rates of complete remission, partial remission, and disease stability were included in the tumor response.
- Tumor markers. Fasting 5 ml blood sample was centrifuged to separate the serum and measured by enzyme-linked immunosorbent assay (R&D; USA) for serum levels of carcinoembryonic antigen (CEA), cancer antigens (CA) 153 (CA153), CA125, and CA19-9.
- Immune function indicators, including CD3^+^, CD4^+^, CD8^+^ . Anticoagulated peripheral blood was collected and peripheral blood mononuclear cells were separated by density gradient centrifugation. Fluorescently labeled anti-CD3 (UCHT1, BD Biosciences, USA), anti-CD4 (SK3, BD Biosciences, USA) and anti-CD8 (SK1, BD Biosciences, USA) antibodies (BC6800, Mindray, China) were added and incubated at 4°C in the dark for 30 minutes. After washing with phosphate buffer solution, the cells were detected using a flow cytometer (BC6800, Mindray, China).
- Blood levels of **cyclooxygenase**-**2** (**COX**-**2**), Decoy receptor 3 (**DcR3**), and Caspase-3 levels were measured by enzyme-linked immunosorbent assay (R&D; USA).
- Toxic side effects, such as bone marrow suppression, leukopenia, vomiting, nausea, and gastrointestinal reactions were recorded according to National Cancer Institute’s Common Terminology Criteria for Adverse Events.^12^

### Statistical Analysis:

All data analyses were conducted using SPSS 25.0 software (IBM Corp, Armonk, NY, USA). The measurement data were represented by mean ± standard deviation. Independent sample t-test was used for inter group comparison, and paired t-test was used for intra group before and after the treatment. Count data were represented by number of cases and analyzed using Chi square test. *P*<0.05 was considered statistically significant.

## RESULTS

This study included a total of 150 BC cases. Among them, 77 patients received Pyrotinib combined with Trastuzumab, and 73 patients received Trastuzumab alone. There was no significant difference in general information such as age, disease stage, pathological type, ECOG score, family history, and hormone receptor status between the two groups (*P*>0.05) Table-I. The tumor response rate of the combined group (94.81%) was higher than that of the Trastuzumab group (84.93%) (*P*<0.05) Table-II.

**Table-I:** Comparison of Patient characteristics between Two Groups.

Patient characteristics	Combined group (n=77)	Trastuzumab group (n=73)	t/χ^2^	P
Age (year)	58.78±9.4	59.85±8.85	-0.716	0.475
***Disease staging [n (%)]***				
Ⅱb	39 (50.65)	38 (52.05)	0.543	0.762
Ⅲa	23 (29.87)	24 (32.88)		
Ⅲb	15 (19.48)	11 (15.07)		
***Pathological type [n (%)]***				
Infiltrating ductal carcinoma	71 (92.21)	69 (94.52)	0.398	0.819
Invasive squamous cell carcinoma	2 (2.60)	1 (1.37)		
Infiltrating lobular carcinoma	4 (5.19)	3 (4.11)		
***ECOG score [n (%)]***				
0	21 (27.27)	17 (23.29)	0.497	0.780
1	42 (54.55)	40 (54.79)		
2	14 (18.18)	16 (21.92)		
***Family history [n (%)]***				
Yes	2 (2.60)	1 (1.37)	0.288	0.591
No	75 (97.40)	72 (98.63)		
***Hormone receptor status [n (%)]***				
Positive	41 (53.25)	37 (50.68)	0.099	0.754
Negative	36 (46.75)	36 (49.32)		

T-test was used for age, and Chi-square test was used for the other characteristics.

**Table-II:** Comparison of tumor response between two groups.

Group	n	Complete remission	Partial relief	Stable disease	Disease progression	Tumor response
Combined group	77	27 (35.06)	32 (41.56)	14 (18.18)	4 (5.19)	73 (94.81)
Trastuzumab group	73	13 (17.81)	32 (43.84)	17 (23.29)	11 (15.07)	62 (84.93)
*χ^2^*						4.059
*P*						0.044

Before treatment, there was no significant difference in serum CEA, CA153, CA125, and CA19-nine levels between the two groups (all *P*>0.05). After treatment, serum levels of CEA, CA153, CA125, and CA19-9 in both groups were significantly reduced compared to before the treatment, and were significantly lower in the combined group compared to the Trastuzumab group (all *P*<0.05) Table-III.

**Table-III:** Comparison of tumor markers between two groups before and after treatment.

Time	Group	n	CEA (g/L)	CA153 (U/ml)	CA125 (U/ml)	CA19-9 (U/ml)
Before treatment	Combined group	77	14.14±2.36	69.02±7.30	86.89±6.82	60.23±5.53
	Trastuzumab group	73	13.57±2.57	70.86±7.71	85.89±8.51	59.13±6.41
	*t*		1.406	-1.498	0.728	1.124
	*P*		0.162	0.136	0.468	0.263
After treatment	Combined group	77	3.69±1.67^a^	29.85±3.92^a^	34.62±5.38^a^	16.92±3.09^a^
	Trastuzumab group	73	5.92±1.55^a^	35.68±5.13^a^	43.18±7.22^a^	23.30±3.94^a^
	*t*		-7.173	-7.831	-6.502	-10.972
	*P*		<0.001	<0.001	<0.001	<0.001

***Note:*** Compared with before treatment in the same group,

a P<0.05.

Before treatment, levels of CD3^+^, CD4^+^, and CD8^+^ were comparable in the two groups (all *P*>0.05). After treatment, the levels of CD3^+^, CD4^+^, and CD8^+^ in both groups were significantly reduced compared to before treatment, but the combined group was higher than the Trastuzumab group (all *P*<0.05) (Table-IV).

**Table-IV:** Comparison of immune function between two groups before and after treatment.

Time	Group	n	CD3^+^ (%)	CD4^+^ (%)	CD8^+^ (%)
Before treatment	Combined group	77	56.51±5.63	36.42±5.51	27.24±3.91
	Trastuzumab group	73	57.61±6.77	35.97±4.99	28.12±4.36
	*t*		-1.088	0.530	-1.296
	*P*		0.278	0.597	0.197
After treatment	Combined group	77	53.81±6.01^a^	34.24±5.09^a^	33.03±4.13^a^
	Trastuzumab group	73	51.71±5.79^a^	31.71±4.69^a^	29.29±3.82^a^
	*t*		0.560	3.162	5.742
	*P*		0.031	0.002	<0.001

***Note:*** Compared with before treatment in the same group,

a P<0.05.

Before treatment, there was no significant difference in the expression levels of COX-2, DcR3, and Caspase-3 between the two groups (*P*>0.05). Treatment led to a significant reduction in COX-2, DcR3, and Caspase-3 levels in both groups. Post-treatment levels of these indexes were significantly lower in the combined group compared to the Trastuzumab group (*P*<0.05) (Table-V). There was no significant difference in the occurrence of toxic side effects between the two groups (*P*>0.05) (Table-VI).

**Table-V:** Comparison of COX-2, DcR3, and Caspase-3 before and after treatment between two groups.

Time	Group	n	COX-2	DcR3	Caspase-3
Before treatment	Combined group	77	123.10±19.69	118.12±18.59	121.43±19.33
	Trastuzumab group	73	121.77±21.96	116.95±20.36	119.66±21.21
	*t*		0.393	0.368	0.535
	*P*		0.695	0.713	0.593
After treatment	Combined group	77	64.33±8.83^a^	44.94±9.42^a^	59.06±13.75^a^
	Trastuzumab group	73	77.19±9.36^a^	51.21±14.02^a^	80.65±21.21^a^
	*t*		-8.651	-3.197	-7.355
	*P*		<0.001	0.002	<0.001

***Note:*** Compared with before treatment in the same group,

a P<0.05.

**Table-VI:** Comparison of incidence rates of toxic side effects between two groups

Group	n	Bone marrow suppression	Leukopenia	Vomiting and nausea	Gastrointestinal reactions
Combined group	77	39 (50.65)	22 (28.57)	29 (37.66)	44 (57.14)
Trastuzumab group	73	42 (57.53)	16 (21.92)	31 (42.47)	44 (60.27)
*χ^2^*		0.243	0.877	0.360	0.152
*P*		0.622	0.349	0.548	0.697

## DISCUSSION

The study showed that Pyrotinib combined with Trastuzumab can effectively reduce serum levels of CEA, CA153, CA125, CA19-9, improve tumor response effect, and is not associated with the increase in side effects in patients with HER-2 positive BC. Our results are consistent with previous research. Xu et al.^13^ confirmed that the incidence of side effects was low when Pyrotinib was used to treat HER-2 positive BC on the basis of capecitabine. Wu et al.^14^ showed that the complete remission rate of HER-2 positive BC treated with Pyrotinib on the basis of docetaxel and Trastuzumab can reach 41.0%, and the tumor response rate can reach 91.6%, without increasing the incidence of side effects. Niu et al.^15^ treated HER-2 positive BC patients with pyrrolidine on the basis of conventional chemotherapy drugs, with a complete remission rate of 30.4% and a tumor response rate of 87.4%. Yan et al.^16^ treated HER-2 positive BC on the basis of capecitabine combined with Pyrotinib, with an objective remission rate of 74.6%, and no treatment-related death events during the treatment. The results of our study further support these reports.

Trastuzumab is a large-molecule monoclonal antibody targeted drug that can block the interaction between HER2 and its ligand, thereby promoting the activation of the signal transduction pathway;^17^ while Pyrotinib is an oral small molecule targeted drug that can easily penetrate the cell membrane and directly act on intracellular targets, blocking the downstream signal transduction by binding to the HER2 receptor to inhibit tumor angiogenesis, thus inhibiting tumor growth.^18^

Levels of T-lymphocytes are good indicators of the immune function status, and lower immune function is associated with higher risk of developing malignant tumors.^19^ The immune function of HER-2 positive BC patients is low, leading to a vicious circle: the immune response system can activate specific T cells, and the presentation of specific lymphocyte antigens can form an immune response, reducing the number of lymphocytes and promoting excessive proliferation of toxic helper cells.^4^,^5^ The current study showed that the Pyrotinib combined with Trastuzumab treatment can more efficiently inhibit the immune response of BC and reduce the damage to the immune function compared to of Trastuzumab alone. The reason may be that when using Trastuzumab alone, although it can block the HER2 signaling pathway and inhibit tumor cell growth, its regulation of immunosuppressive components in the tumor microenvironment is limited. The combination of Pyrotinib and Trastuzumab can more comprehensively block the HER2 signaling pathway, effectively inhibit tumor cell proliferation and survival, and reduce tumor cell secretion of immunosuppressive factors.^4^,^5^,^19^,^20^ This also helps to relieve the inhibition of T cells, resulting in higher activity and quantity of T cells in the combined group compared to the simple group..

Studies have pointed out that apoptosis-related factors are closely associated with the incidence and progress of BC.^19^ COX-2 is an important rate limiting enzyme for prostaglandin synthesis, and its increased expression is positively correlated with BC.^21^ Therefore, blocking COX-2 has great potential in the prevention and treatment of BC. DcR3 belongs to the tumor necrosis factor receptor family, and its expression positively correlates with its ability to bind the ligands and block cell apoptosis.^22^ It promotes the capacity of invasion of BC cells and plays an important role in the metastasis of BC. Caspase-3 is a cell apoptosis-related protease that can affect the cascade reaction of cell apoptosis proteases. It is highly expressed in various cancer types, especially BC.^23^ This study confirmed that Pyrotinib combined with Trastuzumab has high application value in HER-2 positive BC, and can downregulate the expression of COX-2, DcR3 and Caspase-3, and improve the prognosis of the disease. Our results further confirm that the Pyrotinib combined with Trastuzumab can more effectively inhibit growth, metastasis, and invasion of tumor cells.

Xie et al^24^ has shown that Pyrotinib combined with Trastuzumab and chemotherapy offered a promising option with manageable safety profile for HER2-positive metastatic BC. The current study also showed that there was no significant difference in adverse reactions between the two groups, indicating that Pyrotinib enhances the efficacy without increasing the risk of medication. Generally speaking, the adverse reactions of Pyrotinib are mild, mainly diarrhea and skin reactions, which is mild and does not require special treatment.

HER-2 positive BC are prone to cancer cell metastasis and recurrence, which accelerates the progression of cancer and affects the quality of life of the patients. Therefore, blocking the continued growth of tumors and resisting HER-2 expression, thereby inhibiting the continued spread and metastasis of cancer cells, is the treatment principle for HER-2 positive BC. The results of this study may be helpful to guide the treatment of HER-2 positive BC.

### Limitations:

Firstly, this is a single center retrospective study with limited sample size, which may limit the generalizability of the findings. Secondly, the impact of the two methods on the long-term functional recovery of patients was not analyzed. Thirdly, the follow-up and prognosis of the two groups of patients were not analyzed. Further prospective randomized controlled trials with expanding sample sizes are needed to verify our results.

## CONCLUSION

Compared with Trastuzumab alone, a combined regimen of Pyrotinib and Trastuzumab is equally safe, and more effective in regulating expression of COX-2, DcR3, and caspase-3, improving immune function, reducing the level of tumor markers, and improving tumor response in patients with HER-2 positive BC. The findings of this study may be helpful to guide the treatment of HER-2 positive BC.

### Authors’ contributions:

**XY:** Study design, literature search and manuscript writing.

**BT, QM, YT LZ and LX:** Data collection, data analysis and interpretation. Critical Review.

**XY:** Manuscript revision and validation, Critical analysis.

All authors have read, approved the final manuscript and are and responsible for the integrity of the study.
